# Natural Kinase Inhibitors for the Treatment and Management of Endometrial/Uterine Cancer: Preclinical to Clinical Studies

**DOI:** 10.3389/fphar.2022.801733

**Published:** 2022-02-21

**Authors:** Rajeev K. Singla, Sahar Behzad, Johra Khan, Christos Tsagkaris, Rupesh K. Gautam, Rajat Goyal, Hitesh Chopra, Bairong Shen

**Affiliations:** ^1^ Institutes for Systems Genetics, Frontiers Science Center for Disease-Related Molecular Network, West China Hospital, Sichuan University, Chengdu, China; ^2^ IGlobal Research and Publishing Foundation, New Delhi, India; ^3^ Evidence-based Phytotherapy and Complementary Medicine Research Center, Alborz University of Medical Sciences, Karaj, Iran; ^4^ Department of Pharmacognosy, School of Pharmacy, Shahid Beheshti University of Medical Sciences, Tehran, Iran; ^5^ Department of Medical Laboratory Sciences, College of Applied Medical Sciences, Majmaah University, Al Majmaah, Saudi Arabia; ^6^ Health and Basic Sciences Research Center, Majmaah University, Majmaah, Saudi Arabia; ^7^ Faculty of Medicine, University of Crete, Heraklion, Greece; ^8^ Department of Pharmacology, MM School of Pharmacy, MM University, Ambala, India; ^9^ Chitkara College of Pharmacy, Rajpura, India

**Keywords:** hormone-sensitive cancer, metastasis, natural products, medicinal plants, endometrial cancer

## Abstract

Endometrial cancer (EC) is the sixth most prevalent type of cancer among women. Kinases, enzymes mediating the transfer of adenosine triphosphate (ATP) in several signaling pathways, play a significant role in carcinogenesis and cancer cells’ survival and proliferation. Cyclin-dependent kinases (CDKs) are involved in EC pathogenesis; therefore, CDK inhibitors (CDKin) have a noteworthy therapeutic potential in this type of cancer, particularly in EC type 1. Natural compounds have been used for decades in the treatment of cancer serving as a source of anticancer bioactive molecules. Many phenolic and non-phenolic natural compounds covering flavonoids, stilbenoids, coumarins, biphenyl compounds, alkaloids, glycosides, terpenes, and terpenoids have shown moderate to high effectiveness against CDKin-mediated carcinogenic signaling pathways (PI3K, ERK1/2, Akt, ATM, mTOR, TP53). Pharmaceutical regimens based on two natural compounds, trabectedin and ixabepilone, have been investigated in humans showing short and midterm efficacy as second-line treatments in phase II clinical trials. The purpose of this review is twofold: the authors first provide an overview of the involvement of kinases and kinase inhibitors in the pathogenesis and treatment of EC and then discuss the existing evidence about natural products’ derived kinase inhibitors in the management of the disease and outline relevant future research.

## 1 Introduction

Kinases play a significant role in tumorigenesis, cancer cells’ survival, and proliferation. Different studies on human kinases were able to report 555 types of protein kinases based on their sequence analysis and evolutionary history (Baier and Szyszka, 2020). Out of this, 497 were grouped into eukaryotic protein kinases and 58 as atypical kinases. Kinases constitute 2% of the total human genome and the first group of enzymes that play a significant role in oncogenic evolution and are a potential drug target in cancer therapy (Schwartz and Murray, 2011). Mutations and transformations in tyrosine kinases are found to occur in different oncogenic conditions. PI3KCA is a member of the PI3K family with dual specificity of lipid and protein kinases associated with ovarian, breast, endometrial, and colorectal cancer (Gocek et al., 2014). Protein kinases serve as regulators of many cellular pathways which are related to diseases, sometimes as triggers and sometimes as therapeutic mediation points. A growing body of research focuses on natural-source-derived kinase inhibitors as anticancer therapeutics.

Cyclin-dependent kinases (CDK) regulate essential complexes of cell cycle advancement and alteration. In cell cycle regulation, 11 molecules of the CDK family function as leads at three checkpoints: G1, G2, and M checkpoints. CDK4 and 6 D and CDK2-E cyclin regulate G1 and S phase transition, CDK2-cyclin A and CDK1-cyclin B regulate G2, and CDK2-cyclin A and CDK1-cyclin B are also responsible for the phosphorylation of FoxM1 (Forkhead box protein M1) that codes for the transition of a cell from G2 to M phases (Gocek et al., 2014; Finn et al., 2016; Jorda et al., 2018). CDK7 functions as a regulator of different CDKs during different phases of the cell cycle. As CDKs play an important role in ontogenesis in endometrial cancer (EC), CDK inhibitors (CDKin) have a noteworthy therapeutic potential in EC (Sanchez-Martinez et al., 2015). CDKin is classified into 4 different generations based on their development and mechanism of action. The majority of first- and second-generation CDKin have nonspecific action and can produce toxic effects. Dinaciclib, a second-generation CDKin, shows significant activity against the breast cancer cell line by inhibiting Myc. Palbociclib, ribociclib, and abemaciclib are some of the third- and fourth-generation CDKin which are found specific for both CDKs and tumor cell lines (Finn et al., 2016).

Recent studies screening natural compounds as kinase inhibitors indicated particular natural compounds, mostly polyphenols, with a major potential to mitigate kinase mutation. The bioactive compounds directly bind with tyrosine kinases receptors and can regulate many cell signaling pathways by altering their phosphorylation nature.

## 2 Pathophysiology of Uterine Cancer

Unopposed estrogen exposure causes endometrioid tumor development, leading to gland hyperplasia and eventually dysplasia. Intrinsic factors that increase blood estrogen levels, such as obesity and type II diabetes mellitus, are highly associated with endometrial cancer (Onstad et al., 2016). All of them have been highly associated with EC of the corpus (or central) uteri, and in some cases with EC affecting the lower uterine area. Individual subtypes do not have distinctive gross appearances that identify them as such (cell type). The low-volume disease rarely finds residual disease by diagnostic curettage (Ambros and Kurman, 1992). Polypoid nodules and friable nodules develop with localized disease. Widespread involvement of the endometrium, but without a discernible exophytic component, suggests induration of the uterine surface. Necrosis and hemorrhage may be seen. They are characterized as being lighter in color because the areas of myometrial invasion are easily identifiable as light-gray or white zones that stand out clearly from the untreated myometrium. It is usually seen when an expansion of the original lesion is detected.

In addition to extrinsic factors, such as estrogen-only hormone replacement therapy and tamoxifen, intrinsic factors such as inheritance and early puberty can also increase the risk for endometrial adenocarcinoma (Whitney et al., 2004). Endometrial atrophy (reduction in the size of the endometrium) frequently accompanies serous endometrial cancer, and certain genetic anomalies have also been linked to the development of this disease. Estrogen-related cancers are often low-grade and have a favorable prognosis and are known as Type I tumors (Sherman et al., 1995). These endometrial hyperplasia lesions tend to have an immature, undifferentiated look due to the growth of hyperplasia around them. Lopsidedly, 80% of spontaneous tumors are due to them. PTEN and other targets are downregulated, resulting in constitutive activation of Akt and mTOR (Enomoto et al., 1991; Okuda et al., 2010).

Type II tumors are poorly differentiated and are typically seen in older postmenopausal women. When it comes to type II estrogen-independent breast cancers, endometrial atrophy is usually present. There are documented links between ErbB2, P16, and TP53 mutations and the development of type II cancers (Zagouri et al., 2010; Singh et al., 2011). In general, tumors with these features are frequently found to be at an advanced stage or have already metastasized, and this predicts a bad prognosis (Leslie et al., 2005). 6-month survival is quite low in this type of cancer, even with extensive treatment, including chemotherapy and radiation.

Type I and type II carcinomas differ both molecularly and clinically (Lax, 2004). Most type I carcinomas have a modest number of somatic copy number alterations as measured, whereas most type II carcinomas have a large number of them and aneuploidy. Other genes frequently mutated in the process of developing type I tumors include PTEN (about 50% of cases), KRAS (about 20%–30% of cases), ARID1A (a gene encoding a member of the low-grade endometrioid carcinoma subtype—about 40% of this tumor subtype), CTNNB1 (ß-catenin) (approximately 30% of low-grade endometrioid carcinomas), and PIK3R1 (about 20%–45% of cases), whereas mutations of TP53 (approximately 80%–90% of cases), FBXW7 (approximately 20%–30% of cases), and PPP2R1A (approximately 20%–30% of cases) are more frequently found in type II cancers (Tashiro et al., 1997; Kuhn et al., 2012; Matias-Guiu and Prat, 2013; Yeramian et al., 2013). Additionally, 25%–40% of type I carcinomas have a mutant phenotype leading to microsatellite instability (MSI), while this is quite less common in type II carcinomas. MSI typically involves repeated sequences (that is, MSI increases the mutation rate) (Catasus et al., 1998). Also, mutations in PIK3CA are present in about similar numbers in both types of cancer: of these mutations, 15%–20% are found in type I, while the remaining 80%–85% are found in type II (Oda et al., 2005; Bashir et al., 2014). Also, around 30% of grade 3 endometrioid carcinomas include mutations in the TP53 gene (Lax et al., 2000).

It is another example of the importance of risk factors being taken into consideration while doing molecular pathogenesis research. While the constant replacement of the surface epithelium of the ovary may result in p53 mutations piling up in the ovarian or fallopian tube epithelium, it is postulated that p53 mutations accumulate in the surface epithelium of the ovary or fallopian tube, causing genetic harm (Fathalla, 1971; Lee et al., 2007). Conversely, endometrial cancer seems to be without this similar mechanism. “Recurrent uterine lining growth and disruption” may occur, even if the term “incessant ovulation” just means “recurrent menstruation.” In the case of DNA replication errors during cell division, the probability of random genetic mutations developing increases with the number of cell divisions. There are thousands of mutations occurring in each cell cycle to increase the risk of multistep carcinogenesis, yet this may amount to tens of thousands of mutations per gram of proliferative tissue (Moolgavkar and Knudson, 1981; Cairns, 1998). Spontaneous mutations of another tumor-suppressor gene, PTEN, are often seen in the histologically normal endometria of regularly cycling premenopausal women.

The presence of somatically acquired inactivating mutations and/or deletions of the PTEN tumor-suppressor gene in 30%–50% of sporadic endometrial carcinomas favors PI3K/Akt/mTOR signaling in endometrial cancer (Tashiro et al., 1997; Ali, 2000). In the most recent studies, PTEN inactivation has been discovered in up to 83% of the endometrioid endometrial adenocarcinomas (Mutter, 2000). The PI3K/Akt signaling pathway is controlled by the enzyme protein tyrosine phosphatase-T, or PTEN, which inhibits it. PI3K is constitutively active whenever it is activated by mutations that activate PI3K kinase. Downstream pAkt is downstream of Akt, whereas downstream of PTEN is downstream of mTOR. mTOR is a member of the phosphatidylinositol kinase-related kinases. The catalytic activity of an enzyme is regulated by the mitogen-activated protein kinase (MAPK) and the phosphatidylinositol 3-kinase (PI3K)/Akt pathway. mTORC1 activity is regulated by p70S6K and 4E-binding protein 1 (4E-BP1) (Rosenwald et al., 1993).

MSI and mutations in K-ras, B-Raf, FGFR2, PI3K, and beta-catenin may also be found in endometrioid endometrial cancer (Horst et al., 2012; Katoh et al., 2012). This activation of the enzyme K-Ras (which results in an active MAPK) regulates the expression of the proteins ER and beta-catenin, thus controlling their activity. ER actively binds to promoters of pro-growth genes, resulting in higher rates of transcription and, subsequently, cell proliferation. In estrogen-only hormone replacement therapy, the risk of endometrial cancer is greatly increased (Furness et al., 2012).

## 3 Endometrial/Uterine Cancer- Tumor Microenvironment

Tumor cell growth in a cancer patient is influenced by the biological microenvironment inside and outside the cell (Zheng et al., 2021). Factors like mutated p53, PTEN, and KRAS play a crucial role in endometrial cancer progression, but metastasis occurs only after the support of the microenvironment (Richter et al., 2020). Cells like endothelial cells, macrophages, myofibroblasts, and inflammatory cells communicate *via* cytokines, ligands receptors, and growth factors with EC cells and create a suitable microenvironment for tumor cell invasion and metastasis. The factors of microenvironment-derived signals and stromal cell-derived proteins are potential targets as biomarkers for the detection of metastatic EC (Cheng et al., 2021).

### 3.1 Function of Stromal Myofibroblasts in the Microenvironment of EC

Stromal myofibroblasts (SMs) are the most dominant contributors in the microenvironment of EC and many other types of the cancer cell. SMs produce an environment of cytokines to support the growth of EC, their mobility, and metastasis. Some studies show that the interaction between SMs and EC is with the help of hepatocyte growth factor (HGF) pathways (Cancer Genome Atlas Research Network, 2017). The pathway begins with the production of HGFs by endometrial myofibroblasts followed by the interaction of HGF with the receptor on EC cells inducing the EC cell’s invasion and metastasis. Cancer-associated fibroblasts (CAFs) are the plastic host mesenchyme fibroblasts present in the microenvironment surrounding the EC tumor cells. These CAFs are activated by tumor cells, which appear like normal tissue-resident fibroblasts with modified functions like cardinal elongated fibroblastic function, and a mesenchymal marker. CAFs support EC growth, tumor progression, and drug resistance development, but they still do not play a direct role in tumor management (Soslow et al., 2019). CAFs take part in a matrix organization, and stromal structure formation is also known as stromagenesis. This stroma functions as a path to control metastasis of tumor cells and their adhesion to normal cells, migration, and invasion. Recent research based on *ex vivo* and *in vivo* studies reported that myofibroblasts stimulate EC progression using stromal-derived factor-1 (SDF-1) for chemoattracting the tumor niche. SDF-1 binds with chemokine receptor 4 (CXCR4) to control normal and malignant cell trafficking. Studies based on qPCR and immunohistochemistry found high-level CXCR4 mRNA in EC tissue samples indicating its involvement in triggering endometrial tumor cell invasion (Soslow et al., 2019).

### 3.2 Function of Macrophages in the Microenvironment of Endometrial Cancer

**FIGURE 1:**
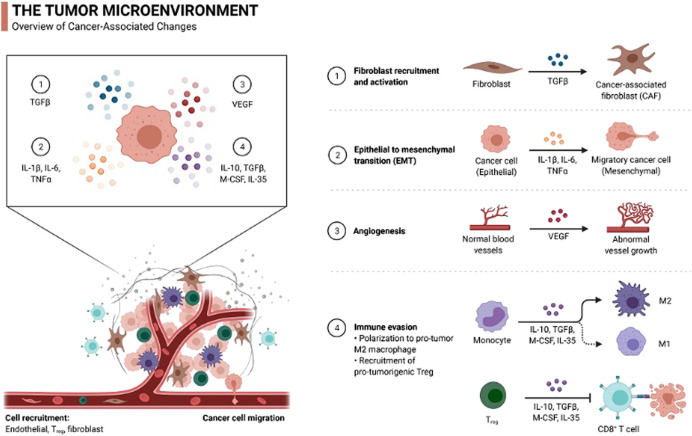
Different types of cells and their function in the EC microenvironment. The microenvironment consists of different components such as myofibroblasts, epithelial cells, and immune cells. (1) TGF-β produced by influx monocytes helps in the conversion of fibroblasts to cancer-associated fibroblasts (CAF). (2) Macrophages induce endometrial carcinogenesis by producing TNF-α, IL-6, and IL-1β that facilitate the conversion of cancer cells to migratory mesenchyme cancer cells. (3) VEGF produced by tumor cells promotes angiogenesis. (4) High-grade EC carries more M2 macrophages, which facilitates more macrophage recruitment *via* blood vessels.

Macrophages, due to their dual function, can either promote the invasion of a cancer cell or inhibit its progression. They are the major component of the tumor cell microenvironment as they release many cytokines, growth factors, and chemokines and also help in EC tumor invasion. The two basic macrophage phenotypes recorded are M1 and M2 (Akbani and Levine, 2016; Cancer Genome Atlas Research Network, 2017). M1 is considered an antitumor with its cytotoxic potential, and M2 is considered a pro-tumor with its tissue and wound healing properties. High-grade EC or type II endometrioid carcinomas carry more stromal M2 tumor-associated macrophages in comparison to type 1 adenocarcinoma. *In vivo* studies have demonstrated that the colony-stimulating factor-1 (CSF-1) when expressed on EC facilitates macrophage recruitment through blood vessels (Yamada et al., 2016). These macrophages remain in the perivascular area of the uterus and induce endometrial carcinogenesis by producing TNF -α (tumor necrosis factor-α), IL-6, (interleukin-6), and IL-1β (interleukin-1 β). These macrophage-derived cytokines help in tumor cell growth and migration to the secondary site (Hu et al., 2016) (Figure 1).

## 4 Signaling Pathways Orchestrating (Coordinating) Endometrial/Uterine Cancer

Endometrial or uterine cancer and its metastasis are under the control of many regulatory signaling pathways. Different signaling pathways in the development of EC identified based on many current studies include the APC/β-catenin pathway based on WNT/β-catenin signaling transduction cascade (Katoh and Katoh, 2007), ErbB signaling pathway (member of the receptor tyrosine kinase family), mTOR/APC signaling pathway, VEGF ligand-receptor signaling pathway, and p53/p21 signaling pathway. These pathways help in escaping apoptosis, inducing cell proliferation, stopping or suspending cell differentiation, and enhancing angiogenesis (Lustig and Behrens, 2003). Here we are discussing some of these signaling pathways in more detail.

### 4.1 Wnt/β-catenin Signaling Pathway

Wnt signaling factors are responsible for cell growth, movement, and differentiation in embryonic growth. Wnt is found to be responsible for the varied signaling cascade activation in tumor formation. Wnt signaling pathways are classified into two types based on the involvement of canonical: canonical and Wnt/β-catenin-dependent signaling pathways and non-canonical Wnt/calcium pathway. β-Catenin-dependent signaling pathways are found associated with human EC/uterine cancer. During the menstruation cycle, the estradiol works as an enhancer for Wnt/β-catenin-dependent signaling pathways, whereas progesterone is found to inhibit Wnt signaling by FOXO1 and DKK1 expression (Jenwitheesuk et al., 2017). The activation of the Wnt signaling pathway causes endometrial hyperplasia resulting in EC.

### 4.2 p53 and p16 Signaling Pathway

Tp53 and p16 are two genes connected with apoptotic disorders significant in many human cancers (Markowska et al., 2014). p53 is related to repairing the damages in DNA, and any mutation in Tp53 results in abnormal p53 protein production which leads to uncontrolled cancer cell growth as found in EC patients. p16 gene mutations change epigenetic modifications and enhance EC carcinogenesis (Kobayashi et al., 2013).

## 5 Natural Kinase Inhibitors for Management of Endometrial/Uterine Cancer- Preclinical Studies

Since ancient times, several medicinal plant extracts and their active components have been demonstrated to have potential uses as anticancer agents (Shen and Singla, 2020; Madaan et al., 2021; Marzocco et al., 2021; Singla, 2021; Singla et al., 2021c; Singla et al., 2021d). Over the last three decades, drug discoveries based on natural products have received considerable interest (Singla et al., 2020a; Singla et al., 2020b; Bansal et al., 2021; Singla et al., 2021b; Sultana et al., 2021). An investigation indicates that a minimum of one-third of the marketed drugs either originated or were derived from different natural resources. Numerous structures of natural product-derived drugs have comprehensively been reported in the literature (Lagana et al., 2019; Singla and Dubey, 2019; Singla et al., 2019; Singla et al., 2021a). Paclitaxel (from the *Taxus* species), camptothecin (from Camptotheca acuminata Decne.), and vincristine and vinblastine (from Catharanthus roseus (L.) G. Don) are some of the most common plant-derived anticancer products. Besides, many other phytochemicals exhibited remarkable effects against various types of cancer. They show such effectiveness by alternating the cancer initiation, development, and progression as well as interrupting several mechanisms like differentiation, cellular proliferation, angiogenesis, apoptosis, and metastasis (Dutta et al., 2019; Gouveia et al., 2019).

A vast number of phytochemicals and natural products demonstrate efficacy *via* numerous signaling pathways to exert tumor inhibitory and antiproliferative effects. Curcumin, caffeic acid, viscolin, pulchranin, retinoids, resveratrol, and guanidine alkaloids exhibit anticancer potential through the regulation of activator protein-1, along with other mechanisms. Many oncogenic transcription factors, such as NF-κB, FoxM1, HIF1, Wnt/β-catenin, and Hh/GLI, are targeted by several potent natural structures. Some of these natural compounds include celastrol, oleanolic acid, ursolic acid, boldine, emodin, resveratrol, genistein, dentatin, garcinol, curcumin, and epigallocatechin gallate (Tewari et al., 2019).

Kinase proteins play a vital role in metabolism, cell signaling, protein regulation, cell trafficking, secretion processes, and many other cellular pathways. Their prominent physiological role is reflected in pharmacological research, where kinase-associated preparations can be found in up to one-quarter of contemporary drug discovery. Among them, the key players are PIK3CA, BRAF, and epidermal growth factor receptor (EGFR), which activate various tumor cell signaling pathways in endometrial cancer cells (Bhullar et al., 2018). Cyclin-dependent kinase Cdc2 (cell division cycle 2), also known as Cdk, is an important regulator at G2/M transition as one of the major checkpoints in the cell cycle. Wee1 kinase and Cdc25C phosphatase are responsible for the regulation of the activity of Cdc2 by phosphorylation and dephosphorylation. Cdc2 binds to cyclin B1 to form a complex that is activated at the onset of mitosis by dephosphorylation of the inhibitory sites on Cdc2 by functional Cdc25C. p21^WAF1/CIP1^, a member of the CDK inhibitor (CDKI) family, can be bound to cyclin B1–Cdc2 complexes and inactivate it.

**FIGURE 2:**
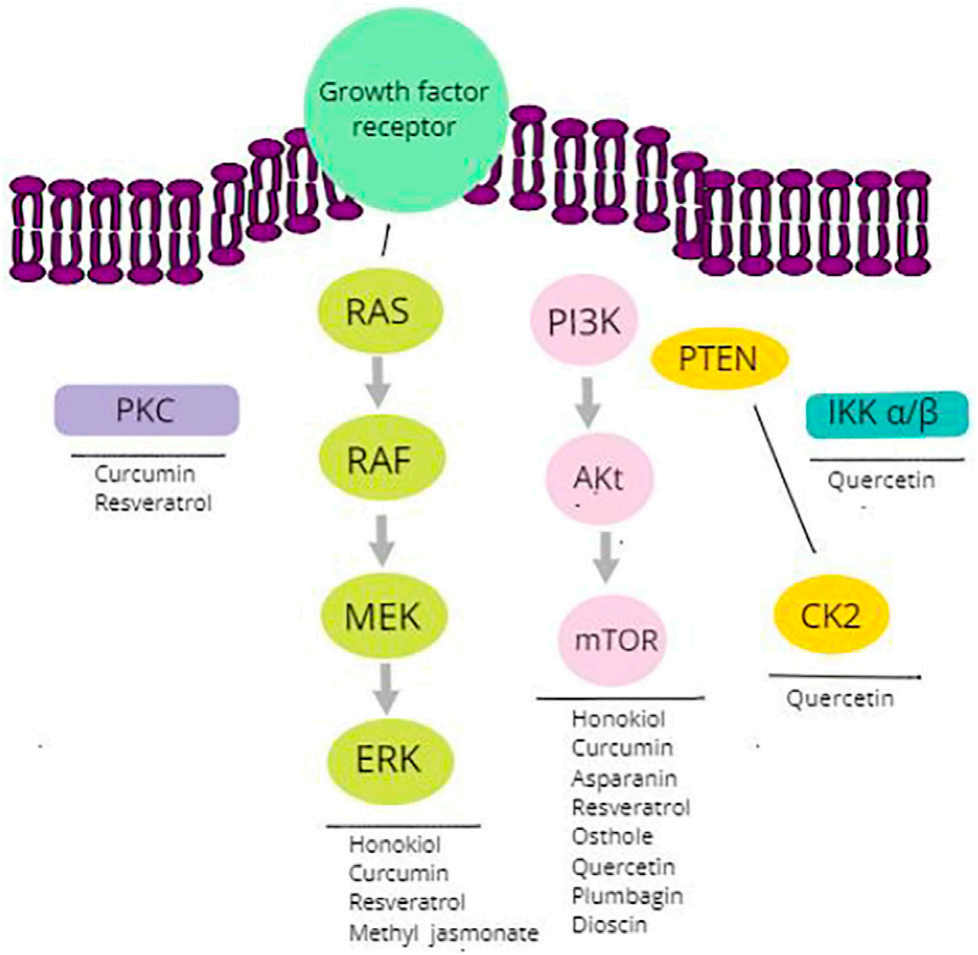
Intracellular signaling pathway, including protein kinases and natural products.

Therefore, natural kinase inhibitors (Figure 2 represents some of the key examples) for the management of endometrial/uterine cancer are being discussed below.

### 5.1 Phenolic and Polyphenolic Compounds

#### 5.1.1 Flavonoids

Quercetin is a wildly distributed flavonoid present in a variety of fruits, vegetables, leaves, seeds, and grains. Prior studies have shown its cytotoxic activity against several cancerous cell lines including skin, lung, colon, and breast (Tang et al., 2020). The growth of endometrial cancer cells (Ishikawa cell line) was treated by quercetin (1, 10, and 100 μM) and inhibited by 3%, 51%, and 87%, respectively. It could also significantly reduce the gene and protein expression of EGF and cyclin D1 at 100 μM. In another study, it was reported that phosphatidylinositol 4-kinase (PI kinase) and phosphatidylinositol 4-phosphate 5-kinase (PIP kinase) were inhibited by quercetin (Kaneuchi et al., 2003). Kinetic analysis has shown that quercetin is a remarkable ATP competitive CK2 inhibitor. Some other kinases such as MEK-1, GSK-3, Hck, and IKKα/β are also inhibited by quercetin at very low concentrations (Hou and Kumamoto, 2010; Baier et al., 2017).

Eupatilin (5,7-dihydroxy-3′,4′,6-trimethoxyflavone), an O-methylated flavone mainly found in *Artemisia* plants, can enhance the activation and phosphorylation of Chk2/Cdc2 checkpoint proteins causing cell cycle arrest in endometrial cancer cells. In addition, eupatilin regulated the phosphorylation of protein kinases ERK1/2, Akt, ATM, and Chk2. It can inhibit the cell growth of Hec1A and KLE (human endometrial cancer cells) with IC_50_ values 82.2 and 85.5 μM, respectively, more potent than that of cisplatin (IC_50_ = 225.6 and 186.3 μM) (Cho et al., 2011; Nageen et al., 2020). The IC_50_ of jaceosidin, another abundant flavone in *Artemisia* species, in Hec1A and KLE cells was 70.54 and 147.14 μM, respectively, which was also markedly lower than that of cisplatin. It has been demonstrated that jaceosidin induces growth inhibition in human endometrial cancer cells by increasing the population of cells in the G2/M phase of the cell cycle as well as eupatilin. It can increase p21 expression in Hec1A cells and induce cell cycle arrest by ATM phosphorylation. Moreover, the cell cycle arrest in the G2/M phase is a result of phosphorylation of the Chk1 and Chk2 proteins, and subsequently phosphorylation and inactivation of Cdc25C.

#### 5.1.2 Stilbenoids

Resveratrol has stilbenoid chemistry which is produced by more than 70 species of plants in response to stress, injury, infection, or ultraviolet radiations (Sirotkin, 2021). Resveratrol was found to have an apoptotic induction in uterine cancer cells. It has been shown that at a low dose of 10 μM, it can increase apoptosis in HeLa, EN-1078D, HEC-1A, and RL95-2 cells. However, it was revealed in Ishikawa cells at a much higher concentration. Resveratrol showed variable effects on protein kinase C *in vitro* (Sexton et al., 2006). It has a strong inhibitory activity on PKCα isomers (IC_50_ = 2.0 μM), whereas other isomers are not affected by resveratrol significantly. It is also described as a moderate inhibitor of several tyrosine kinases and serine/threonine protein kinases, such as Src, ERK1/2, JNK1/2, p38, PKC, PI3K, PKB, and IKK (Slater et al., 2003).

#### 5.1.3 Biphenyl Compounds

Honokiol (3′,5-di-(2-propenyl)-1,1′-biphenyl-2,4′-diol) is a bioactive natural lignan derivative isolated from the leaves and barks of *Magnolia* spp. It belongs to the class of neolignans. Honokiol showed anti-inflammatory, anti-angiogenic, antioxidative, and anticancer activities (Arora et al., 2012). Further research has demonstrated that honokiol is involved in the inhibition of the PI3K/Akt/mTOR pathway and regulation of the EGFR signaling pathway through suppression of EGFR expression and phosphorylation. Moreover, honokiol inhibits the activation of the mTOR *via* inhibition of the ERK1/2 pathway (Banik et al., 2019).

#### 5.1.4 Coumarins

Osthole (7-methoxy-8-(3-methyl-2-butenyl)-2H-1-benzopyran-2-one) is a natural coumarin first derived from *Cnidium* plant. *Angelica*, *Archangelica*, *Citrus*, and *Clausena* are other sources of osthole. Due to its multiple bioactive properties, it has been considered a promising multitarget drug (Zhang et al., 2015). Experiments involving osthole treatment showed that osthole exposure significantly suppressed the growth of human endometrial cancer cell lines (JEC, KLE, and Ishikawa) in a dose- and time-dependent manner, and osthole displayed the most toxicity on JEC cells. Additionally, osthole could increase the expression of PTEN and inhibit PI3K/AKT activity, a key factor in the progression of endometrial cancer, and consequently lead to apoptosis of JEC cells (Liang et al., 2021).

Esculetin is a coumarin derivative that suppresses endometrial cancer proliferation and induces apoptosis by downregulating the BCLXL and XIAP *via* hnRNPA1. Its anticancer actions have been analyzed *in vivo* in nude mouse xenograft models of human endometrial cancer (Jiang et al., 2021).

#### 5.1.5 Other Phenolic Compounds

Psammaplin A is a natural histone deacetylase inhibitor, extracted from the sponge’s species, i.e., *Poecillastra sp*. and *Jaspis sp*., which causes the cell cycle arrest and precipitates cell apoptosis in uterine tumor cells, accompanied by the activation of p21WAF1 and downregulation of pRb and cyclins/CDKs *via* the p53-independent pathway (Ahn et al., 2008).

The action of psammaplysene A on cell proliferation and viability has been investigated by using the BrdU incorporation and cell viability assay, respectively, which showed that it induces cell apoptosis in uterine tumor cells *via* FOXO1 (Berry et al., 2009).

The *in vitro* studies revealed that curcumin prevents the endometrial carcinoma (EC) cells from invading and migrating. It has anti-metastatic properties due to a decrease in the functioning of matrix metalloproteinases (i.e., MMP-2 and MMP-9) which causes the incursion of myometrial carcinoma to the lymph nodes in type-II of EC (Pourhanifeh et al., 2020).

Plumbagin is a naphthoquinone derivative found primarily in medicinal plants and belongs to the secondary metabolites of plants that persuade the arrest of the Ishikawa cell cycle and cell apoptosis by the Akt/PI3K signaling pathway in uterine tumor cells (Zhang X. et al., 2021).

### 5.2 Glycosides

Asparanin A is a spirosteroid saponin (25S-5β-spirostan-3β-ol-3-O-β-Dglucopyranosyl-(1→2)-β-D-glucopyranoside), which is also known as timosaponin A-III, isolated from *Asparagus officinalis* L. root. It has been also isolated from other medicinal herbs *Anemarrhena asphodeloides* Bunge and *Asparagus cochinchinensis* (Lour.) Merr. Asparanin A showed various cytotoxic effects in several cancer cell lines such as liver, colon, and pancreatic cancer. It highly inhibited Ishikawa cell (human EC) proliferation in a time- and dose-dependent manner (IC_50_ = 9.34 μM). It was found to be a very potent reducer of the level of PI3K, AKT, and mTOR pathway in Ishikawa cells, and the expression of *p*-AKT was markedly reduced. Simultaneously, a significant apoptosis induction *via* the mitochondrial pathway has been reported by Asparanin A (Zhang et al., 2019). In a multi-omics study, Zhang *et al.* revealed that apoptosis and autophagy marker proteins including PARP, Bcl-2, AMPK, mTOR, and p62 were notably downregulated, while the cleaved-PARP (c-PARP), ULK1, and LC3II/LC3I were upregulated in Ishikawa cells. It is also found that Asparanin A could induce DNA damage in Ishikawa cells and activate the p53 signaling pathway. The genes ATM, ATR, and CHEK2 were highly expressed and downregulated significantly (*p* < 0.01), which revealed that DNA damage occurred in Ishikawa cells (Zhang F. et al., 2021).

Steroidal saponin and dioscin (diosgenyl 2,4-di-*O*-α-L-rhamnopyranosyl-*β*-D-glucopyranoside) have been isolated from several natural sources, most of which are Dioscoreaceae plants. It is one of the steroidal saponin ingredients from a famous Chinese medicinal plant, *Polygonatum odoratum* (Mill.) Druce (Asparagaceae). Polygonatum is recently considered as a promising new anticancer agent. Numerous steroidal compounds have been reported from Polygonatum with strong immunomodulatory and antitumor activity on different types of cell lines (Yang et al., 2019). Dioscin exhibited the lowest cytotoxicity effects, among them on Ishikawa cells (IC_50_ = 2.37 μM). Previous studies revealed that dioscin can inhibit cancer cell proliferation at different stages through various pathways. It led to the downregulation of Cyclin D, CDK 6, Cyclin A2, Cyclin E, and CDK2 in the G0/G1 phase of the cell cycle through the p21/Cyclin E/CDK2 pathway. Some other signaling pathways such as PI3K/Akt/mTOR and p38/MAPK/JNK are also reported for the anticancer activities of dioscin (Li X. L. et al., 2021). Molecular docking suggests that dioscin can bind to AKT and mTOR to inhibit their phosphorylation (Mao et al., 2020).

Caudatin, a natural glycosidic compound extracted from the Chinese herb “baishouwu,” is a root tuber of *Vincetoxicum auriculatum* (Royle ex Wight) Kuntze that promotes cellular morphological changes and inhibits the proliferation of the cell, formation of colonies, and migration and persuades to apoptosis. The anticancer potential of caudatin has been investigated *in vivo* in HEC-1A and HeLa cell lines by using the MTT assay method on BALB/c mice. Caudatin inhibits the tumor progression by targeting the cytochrome c/caspase and TNFAIP1/NF-κB signaling pathways, as well as reducing the clonogenicity and viability of uterine cancerous cells (Tan et al., 2016).

Chen and his team had evaluated the potential of wogonoside, a potent flavonoid from *Scutellaria baicalensis* Georgi, for the treatment and management of endometrial cancer. They had observed that Wogonoside could upregulate the Mammalian Ste20-like kinase 1, followed by activation of the Hippo signaling pathway. Wogonoside was also found to potentiate the level of ER stress (Chen et al., 2019).

### 5.3 Alkaloids

Berberine (2,3-methylenedioxy-9,10-dimenthoxyprotoberberine chloride) is currently a well-known dietary supplement with numerous pharmacological activities such as antibacterial, anti-inflammatory, antioxidant, and antitumor activities. It is an isoquinoline alkaloid that is found in the roots, rhizomes, and stem bark of *Berberis* spp. (Mortazavi et al., 2020). Berberine showed remarkable inhibitory effects on cell proliferation in different cancer types through various pathways. It exhibited significant cytotoxic effects on human EC cell lines (AN3 CA, HEC-1-A, and KLE cells) in a time- and dose-dependent manner. With the various concentrations of berberine, the levels of *p*-Akt and Akt were markedly declined in all three cells. These results suggested that berberine certainly can also halt cell cycle progression and trigger apoptosis *via* modulation of the PI3K/Akt signaling pathway in endometrial cancers (Kuo et al., 2015). Experiments have exhibited that berberine can also reduce the HER-2 expression or reduce the phosphorylation on various tumor cells (Pierpaoli et al., 2015).

### 5.4 Terpenes and Terpenoids

Sweet wormwood (*Artemisia annua* L.) is a popular medicinal herb, and it has been used as a remedy for chill and fever in East Asia. Several sesquiterpene lactones such as artemisinin have been isolated from sweet wormwood. Recent studies have shown that artemisinin and its derivatives display strong antitumor effects and apoptosis induction in various human cancer cell model systems such as colon, melanoma, breast, ovarian, prostate, central nervous system, leukemic, renal, and endometrial cancer cells (Wong et al., 2017).

It was reported that artemisin dose-dependently had a remarkable cytotoxic activity against Ishikawa human endometrial cancer cell lines. It can induce a G1 cell cycle arrest of Ishikawa cells by downregulating the transcript and protein levels of CDK2 and CDK4 (Tran et al., 2014; Li D. et al., 2021).

Methyl jasmonate is a methyl ester compound that acts as a plant signal-regulating plant morphogenesis and responses to abiotic and biotic stresses. It was isolated as a fragrant compound present in Jasminum essential oil and other plant species for the first time (González-Aguilar et al., 2006). It exhibited a potent pro-apoptotic effect and significant cytotoxicity in human endometrioid Ishikawa and ECC-1 cell lines (Type I) and uterine serous papillary (USPC-1 and USPC-2; Type II). Methyl jasmonate seems to be involved through the PI3K/AKT pathway in sarcoma cell toxicity (Elia and Flescher, 2008). It also enhanced ERK1/2 activity in breast cancer cells. Several studies have revealed that an overexpression of receptor tyrosine kinase (IGF1R) which mediates actions of insulin-like growth factor 1 (IGF1) is related to endometrial cancer. Since AKT and ERK1/2 play important roles in IGF1R signaling, the apoptotic and anti-proliferative action of methyl jasmonate in endometrial cancer cells can be enhanced by co-targeting the insulin-like growth factor-1 receptor (IGF1R) signaling pathway. In addition, it showed remarkable cytotoxic activity in combination with NVP-AEW541, as a selective IGF1R tyrosine kinase inhibitor (Bruchim et al., 2014).

Curcusone C, a rhamnofolane diterpene, present in *Jatropha curcashas*, was found to significantly produce dose-dependent antiproliferative and apoptotic effects in endometrial cancer Ishikawa and HEC-1A cells. Along with other mechanisms, Curcusone C was observed as a MAPK activator and modulating the MAPK/ERK pathway as ERK inhibitor U0126 could attenuate the effect of Curcusone C (An et al., 2020).

Nerolidol is a well-known sesquiterpene alcohol reported from various aromatic essential oils. Dong and the team had studied its significance in the treatment and management of uterine fibroids (UF) by testing the *cis*- and *trans*-nerolidol in ELT3 cells, a rat leiomyoma cell line. A dose-dependent antiproliferative activity of nerolidol was reported along with induction of cell cycle arrest in the G1-phase, through the modulation of the ATM/Akt pathway, downregulation of cyclin-dependent kinase 4 (CDK4), and CDK6 protein expression (Dong et al., 2021).

Hinokitiol showed significant anti-proliferative activity against human endometrial cancer cell lines (Ishikawa, HEC-1A, and KLE). The IC_50_ value was 13.33 μM in the Ishikawa cells, 49.51 μM in the HEC-1A cells, and 4.69 μM in the KLE cells (Chen et al., 2021). Zhang and the team had studied the antiproliferative effects of garcinol against endometrial cancer cells (Ishikawa (ISH) and HEC-1B cell lines). They had reported that garcinol was exhibiting dose-dependent activity with cell cycle arrest at the G1 phase and G2/M phase, by downregulating CDK2, CDK4, cyclin D1, and cyclin B1 as well as upregulating phosphorylated c-JUN N-terminal kinase (JNK) and p-c-JUN (Zhang et al., 2020).

### 5.5 Miscellaneous Compounds

Pheophorbide A is a natural compound that is extracted from *Scutellaria barbata* D. Don, a traditional Chinese medicine. It has been found to exhibit a dose-dependent growth inhibitory impact on human endocrine sarcoma cell lines (MESSA) (Mansoori et al., 2019).

## 6 Clinical Studies-Bench to Bedside: Assessment of Translational Potential of Natural Products to Treat Endometrial/Uterine Cancer

EC, which arises from the uterine lining, is one of the most common gynecological cancers. Various techniques to treat endometrial cancer have been explored throughout the years, among which natural herbal treatments are now proving to be a beneficial strategy (El Khoury et al., 2019). Scientific evidence revealed that phytochemicals have been found to exhibit substantial anticancer potential. Preclinical screening models have been resulting in potential lead compounds for the development of anticancer drugs through pervasive information about preliminary efficiency, pharmacokinetics studies, toxicity profile, and safety measures, which aid to determine whether a compound ought to be taken further for clinical trial purposes in bench-to-bedside processes of drug development (Choudhari et al., 2019).

**FIGURE 3:**
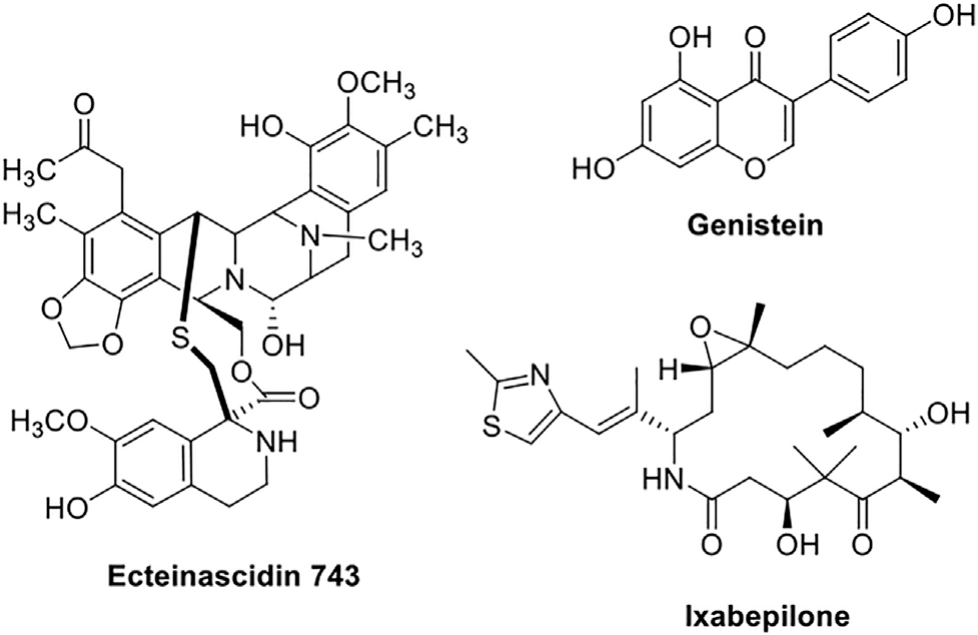
Chemical structures of phytochemicals (Ecteinascidin-743, genistein, and Ixabepilone) used in endometrial cancer in clinical studies.

Ecteinascidin-743 (Figure 3) (ET-743), also known as Trabectedin, is a new marine-derived tetrahydroisoquinoline natural compound, isolated from *Ecteinascidia turbinata* Herdman*.* It binds to the minor groove of DNA in a sequence-specific manner, affecting the transcription-regulated genes selectively. After demonstrating a higher therapeutic index and potency in preclinical research, ET-743 rapidly proceeded to the phase-I clinical trials. In a clinical phase II investigation, ET-743 was shown to be effective as a second-line treatment therapy for individuals with recurrent or persistent EC (McMeekin et al., 2004).

Genistein (Figure 3) is a naturally occurring isoflavone derivative that binds to the endoplasmic reticulum (ER) as an agonist or antagonist, acting as a selective ER modulator having full agonistic activity on the α-receptor and partial agonistic activity on the β-receptor, demonstrating a modulatory action of genistein on cell apoptosis and growth-related pathways in the hyperplastic endometrium. In the presence of a hyperestrogenic environment in premenopausal women with endometrial hyperplasia, genistein exhibited a substantial reduction in the endometrial hyperplasia and associated symptoms (Bitto et al., 2015).

Ixabepilone (Figure 3) is an analog of epothilone B, a natural product. The response rate of ixabepilone in patients with persistent or recurrent endometrial cancer who have progressed despite standard therapy was examined in a clinical phase II investigation. A total of 52 patients were enrolled in this research, with 50 of them being eligible. Ixabepilone exhibited modest activity with short-term duration as a second-line treatment in endometrial adenocarcinoma (Dizon et al., 2009).

## 7 Nano-formulations and Green Synthesis: Strategies to Improve Natural Product’s Pharmacokinetics

No matter how many *in vitro* preclinical studies have shown the potent anticancer capabilities of various natural substances, the findings have been unable to be translated into human trials because of several roadblocks (Leonarduzzi et al., 2010; Jeetah et al., 2014). In the human body, low aqueous solubility, low stability, and poor oral bioavailability are all major problems for natural polyphenolic medicines (Khan et al., 2013; Gohulkumar et al., 2014; Singla et al., 2021c). Because these compounds undergo substantial digestion in the small intestine and liver, only certain conjugates reach the circulation and reach their intended organs. It has been shown that flavonoids can be converted into their various methylated, sulfated, and glucuronidated forms by the phase II enzymes catechol-O-methyltransferases (COMT), SULT 1A1, and uridine diphosphate-glucuronosyltransferases (UDPGTs). However, the potential bioactive properties of these derivatives are still largely unknown. As a result, the quantities of parent chemicals (aglycones) in human plasma are substantially lower than their pharmacologically active levels utilized in preclinical research, staying only in the nanomolar range (Muqbil et al., 2011). Future therapeutic applications of natural polyphenols need the development of innovative drug delivery systems. Nanotechnology has lately emerged as a helpful tool for overcoming the difficulties associated with the *in vivo* use of polyphenols. Nano-sized carriers have been employed to convey parent chemicals directly to the regions where they are required, such as malignant tissues, to demonstrate their powerful anticancer properties to their full therapeutic advantage. To maximize effectiveness and reduce probable systemic damage, tailored treatment may be necessary (Rahman et al., 2014; Singla et al., 2018; Madaan et al., 2021).

The size, shape, and other features of nanoparticles may be tailored to precisely target certain cells. They may be either passive or aggressive in their targeting of the tumor cells. Anticancer medications including nanostructures are manufactured in such a manner that they directly interact with tumor cells *via* ligand–receptor or antigen–antibody recognition. They may interact with cancer cells passively as well. Some researchers have suggested a possible explanation for the preferential accumulation of nanoparticles in solid tumors: There may be abnormal angiogenesis in these tumors that enhances the nanoparticle’s penetration and retention. Targeted administration improves bioavailability and lowers drug concentrations elsewhere in the body, therefore reducing toxicity to a negligible extent.

Additionally, nanoformulations encounter several challenges, including manufacture, distribution, interaction with biological systems, and toxicity (Ahmad et al., 2010; Akhter et al., 2011). In nanoformulation, particle size and size distribution are the most essential properties that affect biodistribution, pharmacokinetics, and safety. While tiny nanoparticles (less than 30 nm in diameter) are quickly eliminated by the excretory system, bigger nanoparticles (more than 200 nm) are taken up by mononuclear phagocytic cells (Desai, 2012). Nanoparticle stability and opsonization are further influenced by the features of the nanoparticle’s surface, including its charge, hydrophobicity, and functional groups (Gessner et al., 2000; Moghimi et al., 2001). As far as nanoformulation is concerned, the main issue is that nanoparticles themselves may be antigenic agents (Trynda-Lemiesz, 2004; Desai, 2012). As a consequence of plasma proteins opsonizing nanoparticles and recognizing them as foreign particles, the complement system may be activated, resulting in life-threatening allergy and hypersensitive reactions, anaphylactic shock, and humoral and cellular immunological responses. It has been shown that nanoformulations may cause thrombogenicity and hemolysis (Dobrovolskaia and McNeil, 2007).

Due to their limited solubility, tyrosine kinase inhibitors (TKIs) have a highly varied bioavailability, which is shown by several characteristics influencing absorption from the digestive tract. In terms of absorption, TKIs are largely reliant on the pH of the solution in which they are dissolved. It is determined whether or not TKIs take on the ionized or nonionized form in the circulation based on luminal pH and pKa, due to their weakly basic nature. Because of the stomach’s acidic environment, the ionized form of the drug dissolves more easily than the nonionized version. Because antacids and PPIs raise the stomach pH, they may reduce the bioavailability of TKIs when used in combination with these medications. Treatment failure and subtherapeutic exposure will occur from a lack of solubility of both forms of the medicine (Augustijns et al., 2014; van Leeuwen et al., 2014; Puszkiel et al., 2017). The passive diffusion of lipophilic and non-polarized TKIs through the lipid bilayer membrane is possible in a normal pH environment. Transmembrane channel-creating proteins carry polarized TKIs, while nonpolarized TKIs do not (Managuli et al., 2018). TKIs have several drawbacks that make them difficult to utilize in clinical practice, and nanomedicine has the potential to overcome these issues. Aside from preserving TKIs from the gastrointestinal tract’s severe environment, nanomedicines increase the intestinal absorption of TKIs and offer TKIs with a regulated release and sustained ionization rate. Nanomedicines are being developed to protect TKIs from the severe environment of the intestines. Even though the majority of TKIs are meant to be taken orally, nearly all of the FDA-approved nanomedicines are designed for intravenous use. Oral nanomedicine delivery presents many unique obstacles; hence, the development of new and better nanovesicles is needed.

Coupling nanotechnology has made it possible to alter the pharmacokinetics and pharmacodynamics of drug molecules. As reported by Han et al., quercitin which is the most abundantly available flavonoid found its use in anti-fibrosis treatment. However, bio-pharmaceutically, quercetin belongs to the BCS Class IV category, thus limiting its absorption. Han et al. prepared quercitin-based nanoparticles using the pulsed laser ablation method with increased solubility and drug release activity (Han et al., 2018). Pharmacologically, nanoparticles showed multifunctional effects *via* inhibition of Aβ aggregation, destabilizing Aβ fibrils, decreasing Aβ-induced oxidative stress and Aβ-mediated cytotoxicity, and opening new channels for the treatment of amyloid-related diseases. Another group of researchers increased the bioavailability of quercitin *via* preparing nanosuspensions consisting of stabilizers such as d-alpha-tocopherol acid polyethylene glycol succinate (TPGS)/soybean lecithin and metabolism inhibitors, i.e., piperine and sodium oleate (Li H. et al., 2021). The presence of stabilizers and metabolism inhibitors increased the oral bioavailability as evident by *in vivo* studies in animal models in comparison to traditional nanosuspensions.

Resveratrol’s poor aqueous solubility and light sensitivity are a major hurdle in its therapeutic efficacy. On exposure to light, the *trans*-isomer gets isomerized to *cis*-form which is less pharmaceutically active. Also, it has poor bioavailability due to pre-systemic metabolism. To tackle this, Peñalva et al. reported a formulation containing casein nanoparticles entrapping resveratrol for increasing its oral bioavailability with a payload of 30 μg/mg nanoparticle (Peñalva et al., 2018). The *in vivo* results showed zero-order release from nanoparticles and release was independent of pH conditions of gastro environment and release the resveratrol in the intestinal region thus decreasing the rate of pre-systemic metabolism.

Honokiol has poor aqueous solubility acting as a rate-limiting step in its biological efficacy. Researchers developed nanoparticles based on honokiol, using the liquid antisolvent precipitation technique which reduces the particle size of molecules and increases their aqueous solubility (Wu et al., 2018). The nanoparticles were prepared using the vacuum freeze-drying method and using HP-β-CD as a cryoprotectant. The solubility of nanoparticles increased remarkably (46.52 mg/ml in comparison to 0.08 mg/ml of free honokiol). Also, the *in vitro* study showed an increase in drug release from nanoparticles due to their amorphous nature and reduction in size, also on the same context the bioavailability also increased in animal models. Another group of researchers also reported nanosuspension of honokiol and an increase in bioavailability and its application in cardio-cerebrovascular diseases (Han et al., 2014).

Similarly, another group of researchers developed gold nanoparticles for photothermal and chemotherapy based on encapsulation of Eupatilin isolated from *Artemisia argyi* H.Lév. & Vaniot (Sun et al., 2021)*.* The nanoparticles were able to permeate through the cytomembrane and locate themselves near the nucleus. Due to the synergism between NIR laser radiation and gold nanoparticles, the chemo photothermal activity of the molecule was enhanced. Radiation with laser light releases the eupatilin and increases the tumor cell killing ability.

Osthol, a coumarin derivative derived from *Cnidium monnieri* (L.) Cusson, found its use in gynecological disorders. Chemically it is lipophilic and has poor aqueous solubility and bioavailability. To enhance its bioavailability, researchers developed enteric-coated nanoparticles based on Eudragit S100 (Li et al., 2018). The nanoparticles showed an increase in plasma concentration and bioavailability in comparison to that per se. The presence of fluconazole inhibited the CYP3A4 pathway regulators; as a result, the metabolism of osthol is decreased and bioavailability increased. Curcumin has a similar kind of physical properties such as low aqueous solubility and bioavailability, thus limiting its use. Gupta et al. developed curcumin-encapsulated lipid nano-constructs (CLEN) (Gupta et al., 2020). The CLEN complexes showed an increase in solubility by a factor of 1.4 × 10^6^ times in comparison to 11 ng/ml (normal curcumin). Other authors have also documented literature citing the benefits of nano-curcumin (Rahimi et al., 2016; Chopra et al., 2021).

Plumbagin is another natural compound possessing low aqueous solubility found in the Plumbaginaceae family. It mainly found its role in anticancer, antidiabetic, anti-inflammatory, etc., activities. Chrastina et al. developed nanoemulsions based on Plumbagin for enhancing its anticancer effect on the prostate tumor (Chrastina et al., 2018). Nanoemulsions consisted of oleic acid, polysorbate 80, and plumbagin. The nanoemulsions showed one phase exponential release profile of plumbagin with a half-life of 6.1 h in gastric fluid and 7.0 h in intestinal fluid. Also, plumbagin nanoemulsions showed increased cytotoxicity toward PTEN-P2 cells in comparison to free drugs. Also, another group of researchers developed plumbagin-coupled silver nanoparticles for their anticancer activity on HeLa cells (Appadurai and Rathinasamy, 2015). Plumbagin showed mitotic blockage and inhibition of the clonogenic survival rate along with silver nanoparticles, due to an increase in internalization of plumbagin.

## 8 Future Research Perspectives

Future research ought to emphasize the from-the-bench-to-the-clinic orientation. *In silico*, *in vitro*, and *in vivo* studies need to expand the reported knowledge about the effect of phytochemicals in EC. It is important to investigate how genetic variance, host–microbiome, and tumor microenvironment can affect the response to treatment. Intra-tumoral and inter-tumoral heterogeneity, the ability of tumors to contain and or differentiate toward different subtypes of cancer cells, is an important factor that can spearhead resistance to treatment and downplay the effect of CDKin in EC. Regular tissue and liquid biopsies have made the continuous monitoring of the tumors’ genomic profile possible and can provide explanations in case of decreased efficacy or failure of potent compounds in the treatment of EC. Organoids offer a promising platform for investigating and modulating the tumors’ microenvironment before researching animal models and humans. In the context of clinical trials, future research should investigate the etiology of treatment failure, when it occurs in a significant portion of the participants, to improve the design of future clinical trials and promote patient and tumor-specific treatment. It is also important to investigate when host parameters (microbiome, inflammation, levels of circulating tumor genetic material) affect the bioavailability, distribution, and metabolism of CDKin in the course of treatment.

Beyond precision medicine and translational research in oncology, it is important to associate findings of EC epidemiology studies with the presence of particular natural compounds in the nutrition or the living environment of patients (Chen et al., 2017; Chen et al., 2020). Potential correlations between the presence of particular compounds in areas where the frequency of EC is lower or the prognosis of the disease is better can indicate phytochemicals with major potential for further investigation. Surveying cultural perceptions that can affect the adherence of patients to adjunct treatments based on phytochemicals can also help design real-world clinical studies and interventions.

## 9 Conclusion

**FIGURE 4:**
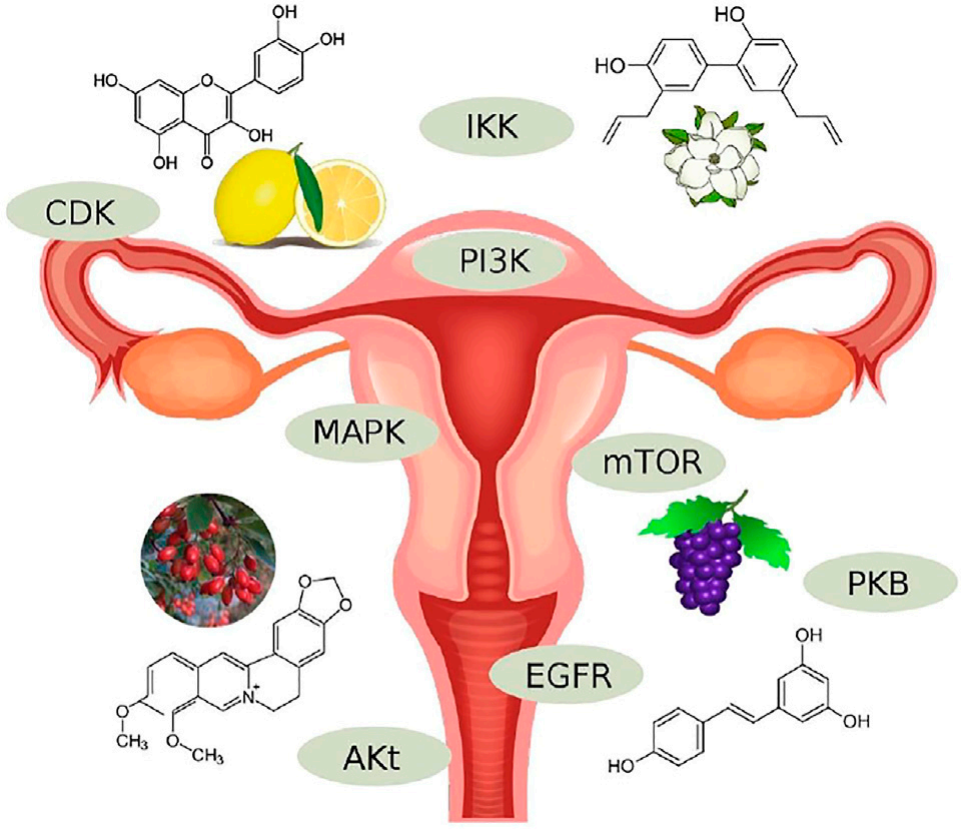
Natural products targeted to protein kinase.

EC is a prevalent type of cancer accounting for significant disease burden, morbidity, and mortality. It is divided into two types. Type 1 is more prevalent, has a better prognosis, and is associated with several mutations and satellite instability mechanisms involving CDKs (PTEN, KRAS, ARID1A, CTNNB1, and PIK3RA1). Therefore, CDKin has major therapeutic potential in the management of the disease. Phenolic and non-phenolic natural compounds (Figure 4) serve as sources of CDKin, which can be applied in clinical practice.

In *in vitro* and *in vivo* studies, flavonoids such as quercetin and eupatilin compete with MEK-1, GSK-3, Hck, IKKα/β, and ERK1/2, Akt, ATM, Chk2, respectively. Stilbenoids such as resveratrol act as moderate inhibitors of tyrosine and serine/threonine protein kinases (Src, ERK1/2, JNK1/2, p38, PKC, PI3K, PKB, and IKK) and exert variable effects on PKC. Biphenyl compounds such as honokiol are involved in the inhibition of the PI3K/Akt/mTOR pathway. Coumarins such as osthole can increase the expression of PTEN and inhibit PI3K/AKT. Glycosides such as asparanin A have an inhibitory effect on the PI3K, AKT, and mTOR pathways, while alkaloids have such an effect on PI3K and AKT. Terpenes and terpenoids can downregulate the transcript and protein levels of CDK2 and CDK4. Phase two clinical trials investigating the effects of trabectedin, a marine-derived tetrahydroisoquinoline natural compound, and ixabepilone, an analog of epothilone B, reported short- and mid-term efficacy of these regimens as second-line treatments. On these grounds, it is a dire need to pursue translational research in the field, mapping the potential therapeutic effect of natural compounds’ derived CDKin and accelerating their proper investigation in clinical trials.
